# What Still Needs to be Noted: Pseudo-Clefts in the Academic Discourse of Applied Linguistics

**DOI:** 10.3389/fpsyg.2021.672349

**Published:** 2021-05-28

**Authors:** Hui Zhou, Ming Chen

**Affiliations:** School of Foreign Languages, Northeast Normal University, Changchun, China

**Keywords:** pseudo-clefts, academic discourse of applied linguistics, generic distribution, discourse functions, evaluation

## Abstract

Pseudo-clefts are the building blocks of coherent discourse progression and serve as a rhetorical toolkit to construct an authorial stance in the academic discourse. Despite an increasing interest in grammatical constructions in the academic discourse, researchers have not treated pseudo-clefts in much detail. This paper explores the features of pseudo-clefts in the corpus of academic discourse in the field of applied linguistics. Here, we take the textual and the interpersonal perspectives, focusing on the use of pseudo-clefts in terms of their distribution in generic structure, discourse functions with reference to clefted constituents, and evaluative meaning. The results show that pseudo-clefts were more frequently used in “Results and Discussion” and Literature Review, performing the functions such as the specification of key terms, generalization of the literature, the description and explanation of findings, etc. They are facilitative in creating information gaps and establishing a logic-semantic expansive relationship between the clauses. The findings also suggest that the pseudo-clefts are evaluative devices and are involved in the construction of authorial identities.

## Introduction

While acknowledging the significant roles that lexical devices take in profiling disciplinary communities, the studies on academic writing have seen the growing attention to specific grammatical resources, and to how they relate to their disciplinary specificity. If lexical devices can be thought to contribute to the identification of specific discourses, such as metadiscursive nouns and lexical bundles in disciplinary writing (Charles, 2007; Hyland, 2008; Jiang and Hyland, 2021), in that certain categories or types of lexical devices are very much constituents of the objects of study in a disciplinary community (i.e., its ontology), grammatical patterns point at the paradigms on which the disciplinary knowledge is based, such as evaluative that-clause (Hyland and Tse, 2005; Kim and Crosthwaite, 2019), it-extraposition (Zhang, 2015), if constructions (Carter-Thomas and Rowley-Jolivet, 2008; Lastres-López, 2020), etc. Given their importance in academic genres and disciplinary discourses, grammatical resources have been studied extensively in English for Academic Purposes (EAP). However, it is surprising that pseudo-clefts remain largely unexplored.

Pseudo-clefts are “divisions of the sentence into two clauses, each with its own verb” (Quirk et al., 1985, 1383), and “all the elements of the clause as a message are organized into two constituents […] linked by a relationship of identity, a kind of ‘equals sign’, expressed by some form of the verb *be*” (Halliday and Matthiessen, 2004, 69). Pseudo-clefts are “tools for presenting and highlighting new information” (Bondi, 2017, 28), serving as the building blocks of a coherent discourse progression, and a rhetorical toolkit to construct an authorial stance, being a grammatical resource for making evaluative meaning. For instance, the sentence, *what cannot be overlooked though is the way in which the successive tasks were introduced and executed*, allows the writer to point to the approach to the tasks and anticipates the possible reaction of putative readers to unfolding texts, i.e., their negligence of the procedure in which *successive tasks were introduced and executed.* Hence, the writer can highlight a critical factor in the process of research in its immediate local context. Compared with its alternative, an unclefted counterpart *The way in which the successive tasks were introduced and executed cannot be overlooked*, the pseudo-cleft sentence has a range of communicative affordances of a highlighted information foregrounded by the WH-cleft (Biber et al., 1999), an identifying relationship between the subject and the complement (Halliday and Matthiessen, 2004), and a dialogic space between the writer and the readers for the sake of its containing an “underlying presupposed question” (Rowley-Jolivet and Carter-Thomas, 2005, 57).

Our interest in pseudo-clefts in EAP concerns their functions at both textual and interpersonal levels in the academic discourse of applied linguistics more specifically. The aim of the present study is to explore the potential of pseudo-clefts in making a coherent discourse, in taking an intersubjective stance and fulfilling evaluative meaning-making functions with reference to the academic discourse of applied linguists. In this way, it sheds light on how the use of pseudo-clefts is related to the discursive nature of applied linguistics (Hyland and Jiang, 2018) and how they construct authorial identities. In practice, the study is motivated as well by the need to design academic writing teaching for a growing number of L2 academic writers in applied linguistics. It can be argued that the evidence from the authentic use of academic language in research articles is needed so as to design the instruction in an effective way. As abovementioned, a considerable and growing body of research on lexical and grammatical devices in the published disciplinary academic writing has greatly strengthened the importance of such an investigation and thus inspired L2 academic writing teaching. However, much remains to be done to analyze the understudied pseudo-clefts in the academic discourse.

In this study, our analyses focus on the overall distribution of pseudo-clefts in generic structures, discourse functions in reference to the syntactically clefted constituents, and the features as evaluative language in a large corpus of research articles in applied linguistics. We seek to address the following research questions:

(1) How do pseudo-clefts distribute in the generic structure of applied linguistic discourse?
(2) What are the discourse functions of the pseudo-clefts?
(3) How do applied linguists use pseudo-clefts to position themselves and their readers?

## Literature Review

Following Weinert and Miller (1996), cleft sentence is a superordinate category under which WH-cleft, IT-cleft, reversed WH-cleft (RWH-cleft), and demonstrative WH-cleft (DWH-cleft) are subsumed, as exemplified in (1)–(4) below.

(1) **What** is important to argue, based on the notion suggested through this study, is that in the course of MRs, learners interact with each other to collectively work on language learning tasks even in situations where no one clearly has great expertise (AL20140103_CO).
(2) It is the variety imparted in elite EM schools that enjoys greater social and professional prestige (AL20140102_LR).
(3) For Schmidt (1990), intake is **what** learners consciously notice or…(MLJ20150103_LR).
(4) this is **where** students carry out an action due to personally related reasons and a desire to attain a valued goal (TESOL20160103_LR).

The complement *that*-clause in the sentence (1), and the subjects in (2), (3), and (4), i.e., *the variety imparted in elite EM schools, intake* and *this*, are termed as clefted constituents. The remaining constituent, which precedes or follows the clefted constituents, is the cleft clause, i.e., *What is important to argue*, *that enjoys greater social and professional prestige.*, *what learners consciously notice or.*, and *where students carry out an action due to personally related reasons and a desire to attain a valued goal*.

The clefted constituents serve the highlighting function (Deroey, 2012). According to Quirk et al. (1985, 1385–1386), the subject, the predicate, the object, the adjunct, and the subject clause can be syntactical focuses in the cleft sentences. Accordingly, the subjects in (1) and (2), the object in (3), and the adjunct in (4) are the highlighted items, respectively, if we transform the four sentences into their unmarked word orders. Weinert and Miller (1996, 174) indicate that these clefted constituents are the marked items from the immediate discourse, “exerting a braking and consolidating effect on the discourse.” Particularly, the IT-cleft and RWH-cleft are thematically marked copular structures in terms of Huddleston’s (1984) interpretation. Halliday and Matthiessen (2004, 70) further point out that in cleft constructions, any constituent of the clause can be made to serve the purpose of Theme or Rheme.

Among the above subcategories, WH-cleft, RWH-cleft, and DWH-cleft are pseudo-clefts. Quirk et al. (1985,1387) point out that the pseudo-cleft is “like the cleft sentence proper, the construction [that] can make explicit the division between given and new parts of the communication.” With the construction built by WH-cleft clause and copular verb, pseudo-clefts are usually interpreted from the perspective of information structure and textual coherence. Biber et al. (1999, 896) define them as constructions which “fit in within the context, thereby building a coherent text that conveys emphasis and related stylistic effects.”

In contrast to the reference grammar, usage-based analyses of pseudo-clefts have been conducted from the perspective of pragmatics and systemic functional grammar. Pragmatics studies have distinguished cleft sentences and pseudo-clefts on one hand, and WH-clefts and RWH-clefts on the other hand in specific genres. It is the sphere in which presupposition and specification apply to pseudo-clefts. Being central to the focusing function, in WH-clefts, the clefted constituents present new information (Declerck, 1984), which “specify the variable in the cleft clause” as an instantiation (Weinert and Miller, 1996, 174), while the cleft clauses create an anaphoric effect, presupposing the givenness of information. However, in RWH-clefts, the highlight is on the entities in the immediate situation (quite often, the object noun phrases (NPs) are focused), with the clefted constituents thematized in a marked way, while the cleft clauses are “forward-pointing and often introduce topics or mark an important point for the following discourse” (Weinert and Miller, 1996, 205).

Systemic functional linguists (Halliday and Matthiessen, 2004, 69) consider pseudo-clefts as thematic equatives, because they construct a “Theme + Rheme structure in the form of an equation, where Theme = Rheme.” The copular sets up an identifying clause, with one element (Identifier) being used to define the identity of the other (Identified). By reference to thematic structure and information structure, systemic functional grammar makes a distinction between an unmarked thematic equative and a marked thematic equative. In the unmarked thematic equative [i.e., WH-cleft, as exemplified in (1)], the WH-cleft clause serves the thematic purpose, conveying a “please-pay-attention message” (Weinert and Miller, 1996, 196), and the clefted constituent is the Rheme, allowing for the arrival of new and important information. WH-cleft clause is in a leading-in position, often accompanied by a faster tempo and a tonic reduction than the clefted constituent in which the prominence lies. Weinert and Miller (1996, 197) metaphorically state that WH-cleft is “both a climax of the one and a bridge to the next,” and therefore, generally used in a descriptive and an explanatory context, as for example in academic description of research.

In contrast, the marked thematic equative [i.e., RWH-cleft, as exemplified in (3)] thematizes the clefted constituents, which are sometimes THAT or THIS deictic, and achieves a combined effect of an initial saliency and semantic cohesion pointing backward (Lu, 2003). The WH-cleft clause is Rheme, indicating new information. DWH-cleft is another type of marked thematic equative, as in (4). Definite THAT/THIS deictics label the point of departure of information flow, serving the function of the Theme. Deictics are also the substitution of the previous message, echoing what Weinert and Miller (1996, 181) say “back-pulling power of the RWH-clefts with THAT or THIS.” In this way the audience tends to pick out the prior referent, near or remote, so that DWH-cleft instantiates the generality and the deixis of the discourse. DWH-cleft can often be found in the summary and generalization of the discourse.

Pseudo-clefts are the potential for meaning-making in context. On one hand, WH-cleft clause is “an instance of a structural feature known as nominalization” (Halliday and Matthiessen, 2004, 69) and the nominalization can be either the Theme in unmarked thematic equatives or the Rheme in marked thematic equatives. In the former, the speaker specifies what the Theme is, meaning “I want to tell you that I want to do something,” and nothing else. It adds a semantic feature of exclusiveness. Similarly in the latter, the speaker identifies the Theme with the Rheme. Note that there is also a meaning of exclusiveness “this and this alone” (Halliday and Matthiessen, 2004, 71).

On the other hand, in the thematic or rhematic nominalization, the WH-cleft clause has been construed as a static factuality. According to Halliday and Matthiessen (2004, 227), in an identifying clause, the functions of the Identified and the Identifier would be realized grammatically by the nominal groups. That is to say, the WH-cleft clause has been metaphorically downgraded as an element (nominal group) in the clause. This rank shift manifests the writer’s interpersonal investment in the WH-cleft clause as a constituent with a factual status. Similarly, Weinert and Miller (1996, 185) observe that the proposition in the WH-cleft clause is “presupposed and needs to be accommodated by the hearer into his discourse model as such.” As a result, the speaker directs the audience’s attention to the propositions expressed by the WH-cleft clause and establishes the credibility of that proposition.

Meanwhile, cleft sentences are believed to carry a contrastive effect. Weinert and Miller (1996, 179) once propose that when people assign prominence to the element in a cleft sentence, they are to introduce or reintroduce a topic into the discourse, to maintain the audience’s attention, to anticipate multiple opinions, and to make a contrast with alternative perspectives. Lu (2003) believes that pseudo-clefts can switch the topic of the discourse, highlight the focal message, and enhance a dynamic interaction between the speaker and the audience. Among the functions, highlighting the focal message is to convey contrastive meaning in its local context. Deroey (2012) proposes a functional framework of WH-clefts specific to academic lectures. There are altogether five functions: informing, elaborating, organizing discourse, evaluating, and classroom management. This functional framework pinpoints the interpersonal function of WH-clefts (i.e., evaluating), but it is not explicitly related to contrastive function. Downing and Locke (2015) summarize the discourse functions of pseudo-clefts as introducing new topics, pointing backward, and modifying the previous statements. If the indefinite WH-cleft clauses point forward to the forthcoming specification about something new, the pseudo-cleft introduces a new topic, similar to Weinert and Miller’s (1996, 181) notion of “forward-pointing power;” if pointing backward to what has been discussed before, the pseudo-cleft serves as an anaphoric function; if a contrastive meaning is conveyed in the pseudo-cleft in comparison with the prior statements, it is what Downing and Locke (2015) term as the modification of previous statements, putting a contrastive emphasis on the clefted constituents.

As regards the evaluative meaning of pseudo-clefts, WH-cleft clauses appear to identify, characterize, and assess anaphoric or cataphoric referents through either the authorial evaluation (e.g., *what is interesting here*) or through invoked evaluative interpretation (e.g., *what still needs to be noted*). Many of these WH-cleft clauses also classify the intersubjective stance of the referents either as monoglossic (e.g., *what we see is*, with the presence of authorial intrusion) or heteroglossic (e.g., *what the critique does make obvious is*) or bare assertion (e.g., *what followed was*).

Empirical research has witnessed a frequent use of pseudo-clefts in spoken discourses. For instance, Delin (1989) found that in the entire Lancaster–Oslo Bergen Corpus (The LOB Corpus), the standardized frequency of RWH-clefts in written data was only 0.04 per 1,000 words, while Deroey (2012) reported that in the British Academic Spoken English (BASE) lectures, the normalized frequency of WH-clefts amounted to 1.03 every 1,000 words. A similar finding was yielded by Weinert and Miller (1996) in which the number of WH-clefts and RWH-clefts together reached 1.3 every 1,000 words in spoken data. They attributed their infrequent use in written discourse to the fact that written language is “less ephemeral and does not require as many attention markers for important items” (Weinert and Miller, 1996, 193).

Studies in disciplinary discourse evidenced a disciplinary specificity of pseudo-clefts. Bondi (2017) analyzed *what*-nominal clauses in historic discourse based on a local grammar of evaluative *what*. *What*-nominal clauses were found to be used as a tool for historians to associate themselves with the shift in a spatio-temporal perspective and positions toward the characters and events in history, and to interact with the multiple voices in disciplinary community. *What*-nominal clauses help diversify the points of view in the discourse progression, expand the dialogic space with the historic academic community, and establish the identity of historians.

In a nutshell, previous studies have investigated pseudo-clefts from the perspective of reference grammar, pragmatics, and systemic functional linguistics. However, there is still a need for further evidence of their use in written discourse, especially in the disciplinary discourse. In this context, the present study examines pseudo-clefts in research articles of applied linguistics in terms of their distribution across generic structures, discourse functions, and evaluative meaning-making and identity construction.

## Data and Methodology

Data for the study consist of 227 research articles in six internationally peer-reviewed journals in applied linguistics, namely Applied Linguistics (AL), Modern Language Journal (MLJ), TESOL Quarterly (TESOL Q), Language Learning (LL), Language Teaching Research (LTR), and Second Language Research (SLR). From each issue of these journals published within the period of 2014–2016, we randomly chose 2–3 research articles, which have presented empirical studies in applied linguistics. These research articles were written by either single author or co-authors, and by either English natives or non-natives. After the portable document format (PDF) files were transformed into plain texts, we manually deleted the title, tables, figures, notes, stand-alone citations, and references, and retained the main bodies of the sampled research articles. Overall, the corpus comprises 1,765,944 tokens and 38,439 types.

In the present study, we decided to focus on the research articles of applied linguistics for two reasons. First, the writers who study language, compared with other disciplinary writers, are more aware of the effects of the language use on the readers (Hyland and Jiang, 2020). As a result, they are more likely to manipulate grammatical devices to interact with readers. In other words, research articles in applied linguistics are suitable data set for our observation of pseudo-clefts in the present study. Second, the use of pseudo-clefts varies according to discipline (Bondi, 2017). Therefore, restricting the data set to one particular discipline (i.e., applied linguistics) would minimize the effect of disciplinary variation and reveal how academic writers manage successful communication with readers through this grammatical device.

The preceding section listed WH-cleft, RWH-cleft, and DWH-cleft as subsets of pseudo-clefts. However, Collins (1991), Weinert and Miller (1996), and Halliday and Matthiessen (2004) emphasize that there have been other subsets of pseudo-clefts, sometimes without a WH-word, as in Collin’s examples (5) and (6) below.

(5) All the car needs require a new battery.
(6) The thing the car needs is a new battery.

They are referred to as ALL-cleft and TH-cleft. In a longitudinal study of pseudo-clefts, Traugott and Trousdale (2013) demonstrate that ALL-clefts evolve into pseudo-clefts as a process of constructionalization. Halliday and Matthiessen (2004) cite *the one* as an example of TH-cleft, and in Collins (1991), there list *the thing*, *the place*, *the reason*, *the time*, *the way* + nominal phrases, or clauses as instances of TH-cleft.

To retrieve and label pseudo-clefts in the corpus, we established the following manual coding system (see Table 1).

**TABLE 1:** Coding system of pseudo-clefts.

	**Category**	**Code**	**Example**
Subset	WH-cleft	W	What is interesting about these forms is that both groups showed long-term gains
	RWH-cleft	R	Another important consideration is what schools can do to mitigate the negative consequences of emotion labor for their FL teachers
	DWH-cleft	D	This may be one of the reasons why planning effect in testing contexts differs…
	TH-cleft	T	Organization strategies are the ones that enable learners to retrieve the knowledge needed to learn the material
	ALL-cleft	A	All the instructor needs to do is to saturate reading materials with the target linguistic item
Clefted constituents	Predicate	PR	What we should attempt to do in the future is find out the important learner-internal and learner-external factors
	Subject	SU	What differentiated auditory and visual CF was the amount of linguistic information provided
	Object	OB	What we found in each narrative were interactions between environment and motivation
	Adjunct	AD	This is where EPT fills a gap, as the participants acknowledged
	Subject clause	SC	What becomes clear from the interviewee’s views on means of diagnosis is that it is to a large extent dependent on an interaction between good training, the availability of resources, and experience and expertise
Discourse functions	New topic introduction	NEW	What also deserves a brief mention are the communicative behaviors of students 1 and 12, mostly on account of the fact that their WTC levels indicated in the last 10 min differed quite dramatically
	Anaphoric function	ANA	… (a) story comprehension, (b) story completion, (c) imitation with expansion, and (d) an asking (games) task. What deserves to be mentioned here is his story comprehension tasks
	Modification of previous statement	MOD	In contrast, the most common contextual factor that instructors cited as discouraging their CF was when errors did not interfere with the student’s communicative meaning
Evaluative meaning	Authorial evaluation	AUEV	What is interesting here is the significant increase in metaphors containing error between PET (B1) and FCE (B2)
	Invoked evaluation	INEV	What cannot be overlooked though is the way in which the successive tasks were introduced and executed
	Monogloss	MONO	What we can see here then is a pattern where the level of WTC is on the increase as students get more involved in…
	Heterogloss	HETE	One major difference in Procall is what van Lier (2002) terms triadic interaction
	Bare assertion	BARE	What could be observed during this time was an increase in WTC at the start of a new task…

We took Quirk et al.’s (1985, 1385–1386) classification of clefted constituents, and classified the discourse functions of pseudo-clefts based on Downing and Locke’s (2015) functional taxonomy. We analyzed the discourse functions of pseudo-clefts in relation to their clefted constituents. Drawing on Appraisal theory’s notion of attitude and engagement (Martin and White, 2005), we distinguished two types of attitudinal meaning: authorial evaluation and invoked evaluation. The former refers to evaluative lexis in Bondi (2017), whereas the latter expresses evaluative meaning implicitly flagged or invoked rather than explicitly marked. In addition, we categorized the sources of perspective or intersubjective stance as monogloss (i.e., authorial voice), heterogloss (i.e., multiple voices), and bare assertions (i.e., factual and interpretive statements in the text).

We had a manual reading of pseudo-clefts in the corpus. This process included an intensive, context oriented, manual analysis of each instance of occurrence in the corpus data. To ensure inter-rater reliability, we invited an experienced instructor of L2 academic writing who had published in internationally peer-reviewed journals to code the data. After a training session, the invited rater, well-informed of the coding scheme, independently coded the corpus data and achieved 94.9% of consistency with our results. For the disparity between the rated results, we had a discussion to reach an agreement.

Altogether, we identified 257 pseudo-clefts in the data set, averaging 145.5 cases per 1,000,000 words. Table 2 illustrates the distribution of five subsets of pseudo-clefts. WH-clefts were the most frequent subset, with 100 *what* clauses, 12 *how* clauses, and three *why* clauses. The TH-clefts ranked second, among which *the ones*, *the ways*, and *the reasons* were frequently used. The RWH-clefts were of a similar occurrence to that of TH-clefts, but with more diverse WH-words (*what*, *why*, *when*, *where*, and *how*) than those in WH-clefts. DWH-clefts were also used in the corpus but in a smaller proportion. ALL-cleft was used least of all with only one case in the whole corpus.

**TABLE 2:** Subsets of pseudo-clefts (raw frequency/per 1,000,000 words).

**Subset**	**WH-cleft**	**RWH-cleft**	**DWH-cleft**	**TH-cleft**	**ALL-cleft**	**Total**
Freq.	115/65.1	54/30.6	32/18.1	55/31.1	1/0.6	257/145.5

As shown in Table 2, RWH-clefts have an occurrence of 0.0306 cases per 1,000 words (30.6 cases per 1,000,000 words), which is consistent with a low frequency reported in Delin’s (1989) study on the same subset in LOB written discourse. This infrequent occurrence might be attributed to the characteristic of written language. For one thing, written texts enable readers to reread the earlier texts and reduce the reliance on attention markers, such as pseudo-clefts in the present study (Weinert and Miller, 1996). For another, pseudo-clefts are characteristic of a low information density as a result of their repetitive content and redundant form (Biber et al., 1999; Qiu and Zhang, 2004). That is to say, a massive information load is expected to be encoded with a concise and compact grammatical structure. Hence, pseudo-clefts infrequently occur in academic writing.

## Distribution in Generic Structure

Table 3 shows the distribution of pseudo-clefts in IMRD generic structures. “Results and Discussion” is the section in which pseudo-clefts predominate, followed by Literature Review, Introduction, Conclusion, and Methodology in a descending order of standardized frequencies. In spite of their uneven distribution across part-genres in research articles, they played an important role in constructing content knowledge specific to part-genres and creating a coherent map of the text to indicate logical connections between successive moves or steps.

**TABLE 3:** Distributions of pseudo-clefts in generic structures (raw frequency/per 1,000,000 words).

	**Introduction**	**Literature review**	**Methodology**	**Results and discussion**	**Conclusion**
WH-cleft	11/87.8	22/57.8	8/21.9	70/97.6	7/39.5
RWH-cleft	5/39.9	11/28.9	5/13.7	25/34.8	5/28.2
DWH-cleft	0/0.0	8/21.0	3/8.2	19/26.5	1/5.6
ALL-cleft	0/0.0	0/0.0	1/2.7	0/0.0	0/0.0
TH-cleft	1/8.0	24/63.1	8/21.9	21/29.3	2/11.3
Total	17/135.7	65/170.9	25/68.4	135/188.1	15/84.7

Introduction is the part that mainly establishes an academic context in which the study is situated, indicates an issue of interest in the field of study, and specifies the research purposes. WH-clefts and RWH-clefts are the majorities in the total occurrences of pseudo-clefts in this section. For example,

(7) **What** emerges is a complex interplay of factors, challenging purely biological explanations of L2 acquisition. In the present study, we assessed… Our goal was to assess which factors affected the speakers’ oral production, in particular their level of grammatical and lexical complexity (LL20160203_IN). (8) Within the classroom and in research, second language (L2) learners differ from one another in how well they use various types of linguistic information. One primary goal of L2 acquisition research is to reach a better understanding of this variability, as it is **what** defines adult L2 acquisition and **what** determines its likelihood of success (Bley-Vroman, 1990) (LL20160204_IN).

WH-clefts can separate two rhetorical moves in the “Introduction” section of research articles, as shown in (7) that WH-cleft clause first indicates the knowledge gap in the field of study and then directs the readers to the research focus. In a similar way, two consecutive RWH-clefts in (8) emphasize the significance of a better understanding of L2 learners’ variability, which happens to be one of research purposes, and then highlight the research contribution.

In the section “Literature Review” where the writer gives a synopsis of the extant studies and establishes a niche for the present one, TH-clefts were used in a higher frequency even than that in “Results and Discussion” (63.1 vs. 29.3). They serve a similar function with RWH-clefts, as shown in (9), introducing an understudied but important variable into the research.

(9) One final (for present purposes) point to note about multimodality is **the way** that it is bound up with the concept of design… Design therefore involves the concept of rhetorical strategy… In this article, I focus on students’ use of rhetorical strategies in the design of multimodal ensembles… (TESOL Q_20140401_LR).

We also identified DWH-clefts. Though less frequent than those used in “Results and Discussion” (21.0 vs. 26.5), DWH-clefts in the Literature Review synthesize the previous research on one hand (by deictic *That*), and on the other hand establish the research gap for the following discourse to unfold (by what-nominal clause), as exemplified in (10).

(10) … many questions remain to be answered, and if we wish to have a better understanding of what underlies weaknesses in SFL reading in order to develop diagnostic procedures, relevant feedback, and more appropriate interventions in order to help weak readers becoming stronger readers, then we need to investigate further and more deeply, in order to arrive at clearer answers to at least some of these questions. **That is what** the exploratory project “Diagnosing reading and writing in a second or foreign language” (DIALUKI), to be described in the next section set out to do (MLJ20160406_LR).

RWH-clefts were also frequent in the Literature Review in which the writer specifies key terms, as shown in (11).

(11) Collective intentionality also underlies the subject matter of psychological accounts of social cognition and human development. For example, a key child development milestone is **when**, through eye gazing, infants infer the intentionality of others through intentions they share as they perceive the same things (MLJ20150301_LR).

A methodology presents the research design and describes the research procedures. The clefted constituent of TH-cleft in (12) gives an account of the participant recruitment, and that of WH-cleft in (13) reports the participants’ demographic background in detail. Both pseudo-clefts stress the subject complements and sufficiently document research methods to allow replication.

(12) **The reason** that the intermediate and the advanced non-native participants were not recruited from the same school was that, while few students at the Chinese University had taken the TOFEL and hardly any scored 100 or above, it was difficult to recruit an adequate number of intermediate Chinese students in the American University (AL20160205_ME).

(13) This section presents a sample of participants’ profiles (Table 1), illustrating the danger of assumptions built on one-to-one relationships between ethnicity and language. **What** is evident here is that the three last learners, who would be categorized as belonging to the Xhosa ethnic group, have markedly different linguistic profiles… Zolile, for example,… (AL20160401_ME).

In the section “Results and Discussion,” the writers report the findings in a systematic and detailed way, and discuss the results with reference to those of previous studies so as to reach generalizable conclusions. This rhetorical function of “Results and Discussion” helps explain why the writers used the most pseudo-clefts, particularly the WH-clefts (accounting for almost half of the total occurrences), serving a descriptive and an explanatory function. As the Theme, WH-cleft clauses appeal to the readers’ attention to interesting findings, as illustrated in (14), and to significant results based on statistical data, as illustrated in (15).

(14) In Figure 9, we must first point out that the large drop in the strict error-scoring line from KET (A2) to PET (B1) is not statistically significant, as there were only in fact two metaphors used at this level. **What** is interesting here is a significant increase in metaphors containing error between PET (B1) and FCE (B2) under the strict scoring criteria (*p* < 0.05) (AL20140201_RD).

(15) As shown in Table 3, CSLE, with 732 occurrences, make heavy use of IT. The other two groups use it less frequently, with PSLE yielding 207 and L1 speakers 161 occurrences. CSLE use IT 4.5 times more than L1 speakers and 3.5 times more than PSLE. **What** stands out in the CSLE data is that almost all occurrences of stance markers are IT; the other two groups demonstrate a more diversified phraseology. For instance, PSLE use I guess almost twice as frequently as L1 speakers (41 vs. 23), but the phrase is used only once by CSLE (AL20160302_RD).

From the insignificant *drop* to a *significant increase* in (14), the writer informs readers of the unexpected results and highlights the seemingly contrasting research findings within the single study. In (15), the WH-cleft clause serves as a transition statement, which signals a logical progression, moving from the reports on *IT* results across three groups to the next.

Reversed WH-clefts, referred to as a thematically marked copular structure by Huddleston (1984) and a marked thematic equative by Halliday and Matthiessen (2004), were also frequently used in “Results and Discussion”. They provide specific examples as evidence to explicate and strengthen the writer’s knowledge claims, as instantiated in (16).

(16) One example of discourse that serves to foster an empowered representation of self is **what** we identified as the “bigger person” discourse. In the following excerpt, Regina draws on this discourse, and also incorporates the technique of direct speech to validate her “bigger person” status (AL20160204_RD).

In (16), the writers exemplify *an empowered representation of self* with a RWH. By thematizing the clefted constituent *one example of discourse*, they place an emphasis on the function of “*bigger person*” discourse, i.e., *to foster an empowered representation of self*, and reattach importance to this key notion (i.e., “*bigger person*”).

Demonstrative WH-clefts were prevalent in “Results and Discussion” as well, indicating their summative roles in the foregoing cause-effect analysis. For example,

(17) Consequently, variables co-occurring with a linguistic form being learned would influence its storage, retrieval, and use in communication. In a way, concomitant variables of this kind can prime the use of linguistic forms due to an alignment at different levels. **That is why** writing topics meant to activate different types of contextual knowledge can have such an immediate effect on learners’ language performance as shown by the results of study 2 (AL20150501_RD).

Conclusion is the section in which writers summarize the major findings of the study and give comments on the findings. In the “Conclusion” section, WH-clefts and RWH-clefts were used to restate the major findings and promote the research contribution in (18) and to make suggestions for improvements and formulate directions for future research in (19).

(18) **What** we found in each narrative were the interactions between environment and motivation, which provides a fertile direction for further study (MLJ20150108_CO).

(19) Future research can further explore this construct-context link by tracing changes in multiple pragmatic constructs (Taguchi, 2016). If we find a variation among pragmatic targets, a follow-up question to pursue is **why** such a variation occurs within the same participant group, in the same environment, over the same time period (MLJ20160402_CO).

## Discourse Functions of Pseudo-Clefts

Through a direct interruption into the ongoing discourse, pseudo-clefts assign a marked status to the clefted constituents, which have a bigger impact than a smooth flow of message by a less interrupted sentence (Weinert and Miller, 1996). Table 4 demonstrates the distribution of discourse functions of pseudo-clefts in applied linguistic research articles with reference to clefted constituents.

**TABLE 4:** Discourse functions of pseudo-clefts in relation to clefted constituents.

	**New topic introduction**	**Anaphoric function**	**Modification of previous statement**
Subject	33	26	28
Predicate	1	3	1
Object	22	15	14
Adjunct	32	27	16
Subject clause	15	5	20
Total	103	75	79

As shown in Table 4, the function of introducing new topics is the most common function of pseudo-clefts in the applied linguistic discourse. The vast majority of pseudo-clefts as a new topic introduction are related to a wide range of clefted constituents, in particular subject, adjunct, and object. For example,

(20) **What** could be observed during this time was an increase in WTC at the start of a new task, a fall toward the end of the video …, an increase as the students started speaking, followed by a rather abrupt drop at the end of the task, which coincided with class termination (LTR20160505_RD).

(21) This is particularly important at level B2, as **this is where** learners tend to switch from closed- to open-class items and use metaphor in new ways, which means that errors and L1 influence are particularly likely to occur at this level (AL20140201_RD).

(22) The main selection principle was to have a mixture representing the class composition: Student 1, Boris, is **what** most teachers would describe as a good pupil: he usually pays attention and generally knows the answer to the teacher’s questions (MLJ20140301_ME).

Delin (1990) proposes that writers assign new information to the WH-cleft clauses so as to indicate that this information is going to be interpreted in a specific manner in the forthcoming discourse, and she defines this phenomenon as *novel instantiation*. WH-clefts usually assume the function of a new topic introduction, as in (20). Likewise, the deictic *this* in (21) is followed by an elaboration of the students’ trajectory of L2 development at level B2, and the pseudo-cleft demonstrates clearly the old/new information structure in textual advancement. As such, the writer in (21) justifies their participant sampling and convinces readers of the rigorous research design. In (22), the writer deploys the pseudo-cleft to carry new information out of the need to direct the readers’ attention to the selection principle of a high-level student.

The functions of pointing backward and modifying the previous statements have almost equal proportions (around 30%) of the total number of discourse functions identified in our data. In terms of anaphoric function, pseudo-clefts also make subject, adjunct, and object constituents salient, especially subject clefted constituent as in (23) and adjunct clefted constituent as in (24).

(23) Taken over a period of time, some positions become more dominant in one’s mode of self-representation (Adams, 2011). For example, being a silent student is a positional identity and one of the multiple identities one has. **What** makes a student silent is the positions that the student takes up and the behaviors he or she displays in relation to other people over a period of time (TESOL Q20140402_LR).

(24) In such a framework, intentional learning is based on explicit processing of input material only; intentionally trying to learn something implies an explicit attempt to do so (**this is why** no arrow is included from implicit learning to intentional learning in Figure 1) (AL20160403_LR).

Thematization of WH-cleft in (23) and deixis in DWH-cleft in (24) realize the focusing function of pseudo-clefts, both of which extend backward over several clauses and reactivate old information. These examples support the arguments by Weinert and Miller (1996) that cleft sentences can focus on either new or old information, rather than merely placing an emphasis on the new one.

The modification of previous statements reveals the contrastive nature of pseudo-clefts in the applied linguistic discourse. It is typically realized by subject clefted constituents, as exemplified in (25), and subject clause as clefted constituents, as exemplified in (26).

(25) Classroom teachers like Sarah are increasingly held accountable for the literacy development of their L2 learners and therefore need support to expand their pedagogic knowledge. **What** is still needed is the evidence of students’ capacity to successfully produce the full range of texts needed for curriculum learning in all discipline areas. There is also a need for… (TESOL Q20160401_RD).

(26) …we interpret the dissociation between the naming times and articulatory measures as an index of active L1 suppression. **What** is not yet known is whether the language control observed in an L2 immersion setting interacts in some ways with executive function more generally (LL20160202_RD).

In (25) and (26), the writers make a contrast between what is already there and *what is still needed* and between what is already known and *what is not yet known*, thereby intensifying the pragmatic force of the clefted constituents to expand the discussion of research results and to arouse the reader’s interest in the revised (or supplemented) information. The analyses of modifying function show that there are often, but not always, stressed elements in either the clefted constituents or the WH-cleft clauses, such as *still* in (25) and *not yet* in (26). Note that the parallelism in (25) with *need*, *needed*, and *a need* as a sequence forms a climax in the discussion.

## Evaluative Meaning of Pseudo-Clefts

The distribution of the evaluative meaning of pseudo-clefts in the applied linguistic discourse is depicted in Table 5.

**TABLE 5:** Evaluative meaning of pseudo-clefts (raw frequency/%).

**Authorial evaluation**	**Invoked evaluation**	**Monogloss**	**Heterogloss**	**Bare assertion**
70/27.2%	13/5.1%	16/6.2%	44/17.2%	114/44.3%

Nearly 44.3% of pseudo-clefts could simply be counted as bare assertions, in which they identify the referents with the features that are proved to be “fact,” as indicated in (27).

(27) For example, in (1c) the lexical meaning of the verb *kick* is integrated into the meaning of the construction “X causes Y to move Z,” resulting in the full meaning, “Pat caused the football to move into the stadium by kicking it.” **What** produces this interpretation is constructional meaning rather than lexical meaning (MLJ20160308_LR).

In (27), the proposition is a barely asserted claim, namely bare assertions or categorical assertions following Martin and White (2005, 99). In the bare assertions, there is no overt reference to other voices or interpretations from an alternative point of view. Their proportion corresponds to that of bare assertions in Corpus of Contemporary American English (academic component of COCA-A) in Bondi’s (2017) study of *what*-nominal clauses. This shows that the applied linguistic discourse features an intersubjectively neutral, objective position to a similar extent with the general academia in the use of pseudo-clefts. These pseudo-clefts were employed to express experiential meanings. According to Martin and White (2005, 100), bare assertions are construed by a sense of “taken-for-grantedness.” That is what the characteristic of the presupposition of pseudo-clefts may suffice. These pseudo-clefts assign given information to the propositions, which are treated as not at issue or up for negotiation. Therefore, readers/listeners “share this value position with the writer/speaker” (Martin and White, 2005, 101).

Another feature of pseudo-clefts is their potential to carry a focal point for negotiation with readers and for argumentation with possible divergent viewpoints. This is manifested in the authorial evaluation, invoked evaluation, and heterogloss of pseudo-clefts. As can be seen from Table 5, 70 occurrences of authorial evaluation, 44 of heterogloss, and 13 of invoked evaluation are identified.

Authorial evaluation is quite directive in the feeling, judgment, and appreciation of the proposition in pseudo-clefts. With overtly evaluative lexis, the writer directs the readers to the attitude they are supposed to share. For instance,

(28) **What** is *interesting* about these forms is that both groups showed long-term gains (AL20140101_RD).

(29) **What** may be *problematic* about this type of sentence splitting is that it seems to promote forward extraction (AL20160303_RD).

(30) **What** is also *worth considering* is the reported communicative behavior of individual students in Group 1 (LTR20160505_RD).

Pseudo-clefts in the above examples reinforce either positive or negative evaluation. The attitudinal evaluation is denoted by *interesting*, *problematic*, and *worth considering*. And in the corpus, there have been a wide variety of evaluative lexes, such as *important*, *new*, *clear*, *evident*, *shallow*, *less foregrounded*, *missing*, *striking*, etc. They are sometimes sharpened by intensifiers like *very*, *particularly*, or softened by hedges like *seem to*, *appear to*, and *may be*, etc. The writer is the default source of evaluation, and the readers, whom the evaluation is directed at, are to be construed as sharing certain kind of value position or attitudes with the writer.

Even with the absence of evaluative lexis, the evaluation might be flagged or evoked by the affordance of the non-evaluative lexis in the pseudo-clefts. According to Martin and White (2005, 65), non-attitudinal lexis can infuse circumstantial meanings into its core meaning, thus provoking or inviting evaluative meaning, as exemplified in (31).

(31) As can be seen in Table 4, four different dummy auxiliaries were found in this experiment: *zijn*, *gaan*, *doen*, and *hebben*. **What** *strikes the eye* in Table 4 is that, in all conditions, the frequencies of dummy *zijn* and dummy *gaan* are much higher than the dummy *doen* and dummy *hebben* (SLR20160104_RD).

With *strikes the eye*, an invoked evaluation of the significant differences in frequencies across the four dummy auxiliaries can be recognized by the readers. However, compared with authorial evaluation, there are fewer cases of invoked evaluation in pseudo-clefts in our data. Applied linguists give more prominence to inscribed authorial evaluation than invoked evaluation by employing pseudo-clefts.

Forty-four occurrences of heterogloss demonstrate that pseudo-clefts signal potential diverse viewpoints and negotiate a dialogic space with readers. By engaging an external voice into pseudo-clefts, the writer carefully records and attributes a knowledge claim [as illustrated in (32) and (33)], acknowledges an external voice by establishing its relation with the authorial voice [as illustrated in (34)], and adopts a particular stance toward the attributed claim, as for instance in (35).

(32) Teacher cognition as defined by Borg (2003, 81) is **what** “teachers think, know and believe and the relationship of these mental constructs to what teachers do in the language classroom”(TESOL Q_20150205_LR).

(33) The last example of joint turn construction is **what** Hashimoto (2007) calls interactive clause chaining with the conjunctive particle te, which links multiple clauses (MLJ20140202_RD).

(34) Related to this perspective are **what** Butler (2004) characterizes as norms of recognition, or ways of being and doing that make individuals intelligible to other… (TESOL Q_20140102_LR).

(35) **What** the critique does make obvious, however, is that in order to reclaim the relevance of language teacher cognition research, we need a firmer commitment to understanding those practices of language teaching, … Language teacher cognition research needs to focus more sharply on how… (MLJ20150301_ME).

As shown in the above sentences, pseudo-clefts report how the key linguistic terms are defined by other academics in (32) and (33), and describe how the language phenomenon is viewed by other scholars in (34), and establish relevance with the external comments on the topic of study in (35). In that case, the linguistic terms, phenomena, relations, etc. are to be variously interpreted and discussed, and the authorial voice can be presented as engaging interactively with other members in the disciplinary community.

The colligation of WH-clefts with reporting speech or citation, such as *defined*, *calls*, *characterizes*, and *make obvious* in the (32)–(35), has been noticed by Cheng et al. (2006) and studied by Bondi (2017) as a “redefining” construction, which is also confirmed in the present study. Our finding is also in well accordance with Deroey’s (2012) analytical results of verb phrases in WH-clefts. This may be explained by pseudo-clefts’ potential of “presenting facts” (Deroey, 2012, 116) in academic discourse.

Another type of heterogloss in pseudo-clefts is realized grammatically through the “modals of probability” (Martin and White, 2005, 104), such as *could* in the following example.

(36) This *could* be **why** hyposegmentation in the first-grade Spanish samples only occurred 10% more often than hypersegmentation (AL20140301_RD).

More examples such as *what may be at play here*, *this may well be the reason that*, etc., show the probability in the interpretation of causal relations. The authorial explanation appears to be one view among a group of possible points of view available in the current context, foregrounding the “entertaining” of dialogic alternatives, in Martin and White’s (2005, 111) term. They argue that the subjectivity that therefore emerges makes the proposition contentious, and there is a chance that the readers may not share this value position with the writer (Martin and White, 2005, 104–105).

Meanwhile, introducing the external voice into the text isolates the assessment in the WH-cleft clause (*What is important*) from the authorial evaluation, and presents it as an external interpretation (*Jaffe, 2009*), as illustrated in (37).

(37) **What** is important here is that multilingual learners have access to additional stance-taking resources (Jaffe, 2009), whole new systems of linguistic engagement (AL20160401_RD).

In this way the writer disassociates himself/herself from a commitment to the proposition, places an emphasis on the evaluation in the thematized WH-cleft clause and minimizes subjectivity, hence warranting readers’ attention and alignment with the writer.

Monogloss refers to an overt authorial presence and a greater authorial visibility by using first person pronouns, such as *I* in (38), and their possessive forms, such as *our* in (39):

(38) **What** I found is that the three teachers who had completed a graduate course devoted entirely to pronunciation pedagogy (Tanya, Laura, and Abby) appeared to use a much wider repertoire of techniques than the other two teachers (TESOL Q_20140106_RD).

(39) **What** our data suggest is that, by age six, sign bilingual deaf children have developed a comparable amount of links in their semantic network in ASL (their L1) to hearing children, with similar proportions of paradigmatic and syntagmatic connections (LL20160404_RD).

The authorial voice in the WH-cleft clauses demonstrates an explicit source of information and highlights the writer’s responsibility for the argument and credit for the research outcome. Interestingly, the monogloss in pseudo-clefts occurs mostly in case studies in our data, largely with reference to personal accounts of qualitative research, as illustrated in (38).

As pointed out by Hyland and Jiang (2018), applied linguists tend to avoid using self-mention in comparison with science writers of biology and electrical engineering, so that they seem to be less visible in their academic discourse. In our data, the frequency of monogloss is lagging behind compared with bare assertions and heterogloss, which tempers a less personal stance and confirms a “shift toward a more ‘author evacuated’ style of argument” (Hyland and Jiang, 2018, 28).

Martin and White (2005, 128) define self-mention as formulations of pronouncement, which “set authorial voice against the [heteroglossic] diversity, presenting the voice as challenging or heading off a particular dialogistic alternative.” That is to say, explicit authorial involvement implies the existence of resistance to the authorial voice on one hand, and puts any reader who would pose a question or an alternative position at stake on the other hand. That is how the dialogic space for negotiation between the writer and readers is condensed.

In short, the evaluative meanings of pseudo-clefts facilitate the positioning of writer–reader relationship, and help establish an argumentative space for the negotiation of the writer’s evaluation and stance with putative readers. Furthermore, the qualitative analysis at the interpersonal level also found that pseudo-clefts contributed to the construction of authorial identities or *persona*.

First, an applied linguist is *a researcher* when he/she employs pseudo-clefts as bare assertions of observed results or experimental findings. Second, an applied linguist can also manifest himself/herself in *a reporter* role when the use of pseudo-clefts is for attributing the proposition to external academic sources that often support his/her own academic efforts. This role is named after frequent use of reporting verbs in pseudo-clefts. The reporter role reflects the writer’s expertise in terms of knowledge and competence to establish relevance with diversified perspectives in the disciplinary community. Third, applied linguist also takes on the role of *an evaluator* when the use of pseudo-clefts is for authorial evaluation as well as invoked evaluation. This role reveals the writer’s “awareness of values and positions that may be inscribed in or evoked” (Bondi, 2017, 41) by pseudo-clefts here. Finally, following Fløttum et al. (2006), the identity of *an arguer* is assigned when an applied linguist uses the first person pronoun combined with research-related words, such as research verbs or words related to research activities. The arguer role is the least common role among the authorial identities constructed by pseudo-clefts.

## Conclusion and Implication

We examined the distribution of pseudo-clefts in the IMRD generic structure of applied linguistic discourse and showed how they advance the discourse progression. In Introduction, pseudo-clefts are used to bridge successive moves. In Literature Review, TH-clefts introduce research foci, RWH-clefts specify key terms, and DWH-clefts synthesize the previous research and identify the research niche. In Methodology, pseudo-clefts justify the researchers’ rigorous research design and sufficiently document research methods and procedures to allow replication. In “Results and Discussion” where pseudo-clefts are most frequently used, they report and interpret the research findings in most cases. Also, they provide specific examples as evidence to support the results. In Conclusion, pseudo-clefts summarize the research findings and suggest implications for future research.

Discourse functions of pseudo-clefts were observed to distribute unevenly in applied linguistic discourse, with a new topic introduction being the most salient function and the other two functions (the anaphoric function and the modification of previous statements) being less popular. More specifically, the new topic introduction reveals how the pseudo-clefts build information structure and highlight new information. In contrast, the anaphoric function refocuses the readers’ attention to the aforementioned information and encapsulates the given information. These two functional categories indicate that the pseudo-clefts give prominence to both old and new information in the academic discourse, reflecting a shared rhetorical practice of the discursive nature in soft knowledge fields, such as applied linguistics here. Pseudo-clefts help the writer move back and forth freely in the global discourse and integrate what is already known with what the writer thinks the readers might not but should know, while recognizing their prior knowledge. Vice versa, they also assist readers in tracking information in the text and attending to new topics. Another functional category, the modification of previous statements, marks additive or contrastive relations between the aforementioned claims and the propositions in pseudo-clefts, associated closely with the logic-semantically expansive meaning of pseudo-clefts.

The pseudo-cleft has been shown to be an evaluative and a stance-taking structure in this study. Applied linguists embed their authorial evaluation and viewpoints into this structure, hence aligning their readers into a community of shared evaluation and viewpoints. They also apply pseudo-clefts to establish an objective stance since the majorities of the structure are for barely asserted propositions. Moreover, applied linguists may imply that the proposition in pseudo-clefts is one of many possibilities, hence positioning readers in doubt and to be won over in the negotiation. These findings once again demonstrate the discursive nature of applied linguistic discourse since the writers construct a particular set of dialogic relationships with their readers.

As regards the writer roles, the use of pseudo-clefts has contributed greatly to the authorial identity construction of applied linguists in the corpus. Four types of roles are constructed by pseudo-clefts here, which are researcher, reporter, evaluator, and arguer. Pseudo-clefts are not just indicators of information arrangement for knowledge construction. They also form the bases of shared disciplinary values and foundations for professional identity as an expert member in the academic community.

At a first glance, in the present study, the low frequency of pseudo-clefts in research articles of applied linguistics might seem insignificant and the generalizability is restricted by the analysis of this single grammatical device. However, the infrequent occurrences cannot be seen as evidence to undermine the significance of pseudo-clefts in academic writing. Instead, pseudo-clefts play a crucial role in the negotiation of writer–reader relationships in academic writing. Specifically, by using pseudo-clefts, the applied linguists may take a stance at the way the disciplinary knowledge is constructed and express evaluation toward the clefted constituents and the writer–reader relationships in the discourse. Since there have been a growing interest in the molding of writer–reader relations and evaluative meaning-making in EAP writing (Thompson and Ye, 1991; Hyland and Tse, 2005; Bondi, 2017; Kim and Crosthwaite, 2019), linguistic and pedagogical research alike have recognized the importance of the writer’s ability to use the grammatical and lexical devices to persuade their readers of the knowledge claims they made and to position themselves in relation to alternative viewpoints (Liardét, 2013; Stapleton and Wu, 2015; Lee et al., 2021; Wang and Zhang, 2021). As regards pseudo-clefts in the present study, their meaning-making potentials at both textual and interpersonal levels in academic writing of applied linguistics provide a fuller picture in which how grammatical devices are used to point at the paradigms on which the disciplinary knowledge is based, and to create a range of authorial identities in academic communication.

The findings suggest the necessity for associating pseudo-clefts with appropriate generic structures in order to disclose their usage at the discourse level. The analyses of pseudo-clefts in IMRD structures demonstrate that pseudo-clefts can facilitate the construction of content knowledge in part-genres of research articles, realizing ideational function. The L2 academic writing pedagogy may thereby let the instruction on the use of pseudo-clefts integrated into the genre pedagogy in terms of the patterns of discourse development with the help of pseudo-clefts. It also shows in the present study that pseudo-clefts can manage information flow and realize discourse function of contrasting, which can be considered as textual function. With a manipulation of Theme-Rheme information structure in pseudo-clefts, academic writers may introduce a new topic or summarize the preceding information. With the help of contrastive markers, they may highlight the information that has been contrasted, revised, or complemented in the pseudo-clefts. In L2 academic writing practice, L2 academic writers may associate textual positions in pseudo-clefts (i.e., WH-clefts with wh-clauses in the thematic position, or RWH-clefts and DWH-clefts with wh-clauses in the rhematic position) with the information packaging arrangements in academic writing. Moreover, the findings of the interpersonal functions realized by pseudo-clefts can also be of pedagogical implication to the teaching of how to position the writer–reader relationships and negotiate a dialogic space in academic writing practice. L2 academic writers may inscribe or invoke the evaluative meaning, make a varying degree of authorial intrusion, and take different discursive roles by using pseudo-clefts in academic writing. The three functions of pseudo-clefts correspond to the functions of basic wh-clefts in academic lectures (Deroey, 2012), and these findings about the above functions of pseudo-clefts have some useful implications for the L2 academic writing pedagogy aimed at L2 academic writers generally and L2 learner writers for academic purposes in applied linguistics specifically.

This study adds to a growing area of research into grammatical patterns in the academic discourse and it offers evidence of how disciplinary knowledge of applied linguistics is discursively constructed and dialogically negotiated with pseudo-clefts. However, the limited data from one discipline mean that the findings must be interpreted with caution and these observations might only be applicable to typical social knowledge fields such as applied linguistics.

## Data Availability Statement

The raw data supporting the conclusions of this article will be made available by the authors, without undue reservation.

## Author Contributions

Both authors listed here have made a substantial, direct and intellectual contribution to the work, and approved it for publication.

## Conflict of Interest

The authors declare that the research was conducted in the absence of any commercial or financial relationships that could be construed as a potential conflict of interest.
